# Analysis of the caregiver burden associated with Sanfilippo syndrome type B: panel recommendations based on qualitative and quantitative data

**DOI:** 10.1186/s13023-019-1150-1

**Published:** 2019-07-08

**Authors:** Elsa Shapiro, Charles Marques Lourenço, Neslihan Onenli Mungan, Nicole Muschol, Cara O’Neill, Suresh Vijayaraghavan

**Affiliations:** 1 Shapiro Neuropsychology Consulting, LLC, 820 NW 12th Avenue, Portland, OR 97209 USA; 2 University of Minnesota, Minneapolis, MN USA; 3 Faculdade de Medicina, Centro Universitario Estácio de Ribeirão Preto, Ribeirão Preto, SP Brazil; 4 Cukurova University, Adana, Turkey; 5 University Medical Center Hamburg-Eppendorf, Hamburg, Germany; 6 Cure Sanfilippo Foundation, Columbia, SC USA; 7 Birmingham Children’s Hospital, Birmingham, UK

**Keywords:** Sanfilippo syndrome, Caregiver, Burden of disease, Quality of life

## Abstract

**Background:**

Sanfilippo syndrome type B (Sanfilippo B) belongs to a group of rare lysosomal storage diseases characterized by progressive cognitive decline from an early age, acute hyperactivity, and concomitant somatic symptoms. Caregivers face a unique set of challenges related to the complex nature of Sanfilippo B, but the burden and impact on quality of life (QoL) of caregivers is poorly defined and best practice guidance for clinicians is lacking.

**Methods:**

An international clinical advisors meeting was convened to discuss key aspects of caregiver burden associated with Sanfilippo B based on findings from qualitative and quantitative research undertaken to identify and quantify the nature and impact of the disease on patients and caregivers.

**Results:**

Providing care for patients with Sanfilippo B impinges on all aspects of family life, evolving as the patient ages and the disease progresses. Important factors contributing toward caregiver burden include sleep disturbances, impulsive and hyperactive behavior, and communication difficulties. Caregiver burden remained high throughout the life of the patient and, coupled with the physical burden of daily care, had a cumulative impact that generated significant psychological stress.

**Conclusion:**

A Sanfilippo-specific QoL questionnaire is needed that is directed at caregiver needs and burden and best practice management of these domains.

**Electronic supplementary material:**

The online version of this article (10.1186/s13023-019-1150-1) contains supplementary material, which is available to authorized users.

## Background

Mucopolysaccharidosis type III (MPS III; also known as Sanfilippo syndrome), a group of rare, genetic lysosomal storage disorders, is characterized by a deficiency in 1 of 4 enzymes involved in the degradation of the glycosaminoglycan heparan sulfate, resulting in progressive cell damage and multisystem disease [1–5]. Four subtypes of Sanfilippo syndrome (A–D) have been identified based on the specific enzyme deficiency [1, 2], along with their underlying genotypes [5]. In studies, the incidence of Sanfilippo syndrome subtypes ranges between 0.28 and 4.1 per 100,000 live births depending on geographic region, with types A and B being more common than types C and D [6–8].

Patients with Sanfilippo syndrome type B (Sanfilippo B) are deficient in the lysosomal enzyme, alpha-N-acetylglucosaminidase, which is involved in heparan sulfate degradation [2]. This deficiency causes large amounts of heparan sulfate to accumulate in the cells and tissues of the body [2, 4]. As a result, progressive cellular damage occurs and patients present with a spectrum of symptoms that progress with age and affect multiple organ systems. Brain disease leads to cognitive impairment and ultimately severe morbidity and premature death [4]. Symptoms generally begin between the ages of 2 and 6 years and include slowing in developmental milestones and language acquisition, hyperactivity usually unresponsive to medication, and sleep disorders and disruptive behavior sometimes perceived as aggression that may predate the diagnosis of Sanfilippo syndrome [1, 9].

The progression of Sanfilippo B occurs across 3 loosely defined phases. A period of normal development is followed by various stages of neurocognitive and somatic signs and symptoms that vary in severity depending on disease phenotype [8]. An attenuated phenotype, sometimes presented by patients with Sanfilippo B, involves a slower progression of symptoms and longer lifespan than those with the rapidly progressing form of the disease, for which life expectancy is severely shortened [8, 10]. Mortality has been reported to range from the second to seventh decade of life and pneumonia is a leading cause of death [3, 10]. Early diagnosis in the slow-progressing population is more challenging and patients may remain undiagnosed until adulthood [8]. In a single-center study, diagnosis occurred earlier in patients with the rapidly progressing phenotype of Sanfilippo syndrome (54 months; range, 34–79 months) than in patients with the attenuated phenotype (71 months; range, 20–522 months) [11]. Symptoms of Sanfilippo B can also masquerade as a behavioral disorder. Several reports have shown patients to present with symptoms consistent with a variety of behavioral disorders, including autism and attention deficit hyperactivity disorder, which have resulted in incomplete or delayed diagnosis of Sanfilippo syndrome [5, 12–15].

Care for patients with Sanfilippo B is provided primarily by parents, but siblings, relatives, partners, or professional caregivers may also provide support. [16]. The burden on caregivers has a wide impact on all dimensions of day-to-day family life that extends to the physical and psychological wellbeing of the caregivers. [4, 16]. Caregivers may experience a high physical burden from coping with the effects of behavioral difficulties (eg, hyperactivity and impulsiveness), sleep disturbances, and somatic symptoms (eg, respiratory infections and gastrointestinal disturbances) [8]. Although little research has been published regarding caregiver burden in Sanfilippo B, survey findings of caregivers of Sanfilippo syndrome patients (including those with Sanfilippo B) indicate that they experience a substantial loss of health-related quality of life (QoL) and a high level of anxiety and depression [16, 17].

Because of the progressive nature of Sanfilippo B, caregiver burden is believed to remain high throughout the patient’s lifetime. Shapiro et al. (2015) have shown that the trajectory of Sanfilippo B is associated with increased problems in areas concerning lack of fear, lack of impulse control, and lack of social reciprocity as the patient ages [18]. As the patient ages and the disease advances, the provision and requirements of care change and impact all aspects of family life [4].

Given the challenges and high caregiver burden associated with Sanfilippo B, a meeting of clinical advisors was convened to better understand the family experience of caring for patients with Sanfilippo B and how their experiences have changed and evolved as patients age. The outcomes from this consensus panel discussion are reported here.

## Methods

An international panel of clinical advisors with expertise in the care of pediatric patients with Sanfilippo B and lysosomal storage disorders was convened for a 1-day face-to-face meeting in London, UK. The panel included a pediatric physician, neuropsychologist, endocrinologist, metabolic specialists, and neurogeneticist; one of the participants was also a parent caregiver. The meeting was organized and facilitated by ICON plc (North Wales, PA, USA) and supported by BioMarin Pharmaceuticals Inc. (Novato, CA, USA).

During the meeting, 3 phases of secondary research provided necessary background information for the discussions of the clinical advisors panel to better understand and characterize caregiver burden associated with patients with Sanfilippo B. This secondary research, undertaken by BioMarin Pharmaceuticals Inc., included (1) qualitative research with parents and caregivers, (2) qualitative research with global Sanfilippo B experts, and (3) quantitative research with global clinicians (treaters)/patient advocates for Sanfilippo B (Table 1). The objective of the qualitative research was to provide the clinical advisors with necessary information to assist with their deliberations and recommendations to better understand the family experience of caring for patients with Sanfilippo B, including how caregivers’ experiences change with disease progression, identification of parent and sibling stressors, and to assess the range of experts’ clinical perception of caregiver burden as well as identification of unmet needs in the treatment and care support of Sanfilippo B. A copy of the questionnaire used during the background qualitative research is provided as Additional file 1. The objective of the quantitative research was to measure clinical perception of caregiver burden, understand clinicians’ perspective on the level of caregiver stress and burden, and identify priorities for treatment and caregiver support.

**Table 1:** Phases of Secondary Research

Secondary Research Phase	Research Dates	Method	Participants	Demographics
Qualitative	March and May 2015	60-min telephone interview	26 parents caring for children with Sanfilippo B	9 countries: United States (*n* = 6), United Kingdom (*n* = 5), Spain (*n* = 4), Turkey (*n* = 4), Japan (*n* = 3), Argentina (*n* = 1), Brazil (*n* = 1), Canada (n = 1), and Portugal (*n* = 1)
Qualitative	April and June 2017	45-min web-enabled telephone interview	5 global clinical experts in Sanfilippo B	5 countries: Australia, Brazil, Spain, Turkey, and the United States (*n* = 1 for each country)
Quantitative	July and August 2017	Online survey	46 Sanfilippo clinicians and patient advocates	12 countries: United States (*n* = 10), Brazil (*n* = 9), Colombia (*n* = 7), Argentina (*n* = 4), France (*n* = 3), Turkey (*n* = 3), Germany (*n* = 2), Italy (*n* = 2), Spain (*n* = 2), United Kingdom (*n* = 2), Australia (*n* = 1), and Portugal (*n* = 1) Specialties included geneticist/neurogeneticist (*n* = 17), metabolic specialist (*n* = 12), pediatric neurologist (*n* = 6), neurologist (*n* = 3), pediatrician (*n* = 3), and other (*n* = 5)

## Results

During the meeting, opinion from the panel was obtained in the following areas based on the data presented: source and magnitude of caregiver burden and challenges of early diagnosis, impact of caregiver burden on the QoL of Sanfilippo B families, and mitigating disease burden. Relevant findings provided to the panel from the background qualitative and quantitative research are presented below first, followed by the opinion of the panel. The panel then provided several recommendations based on their discussion.

Among the sample of 26 caregivers, the children with Sanfilippo B had an average age of 9 years (range, 4–50 years), with 9 patients ≤6 years and 21 patients > 6 years; 4 patients had the attenuated phenotype (≥25 years; range, 25–50 years).

### The source and magnitude of caregiver burden and the challenges of early diagnosis

#### Background research findings

**Fig. 1:**
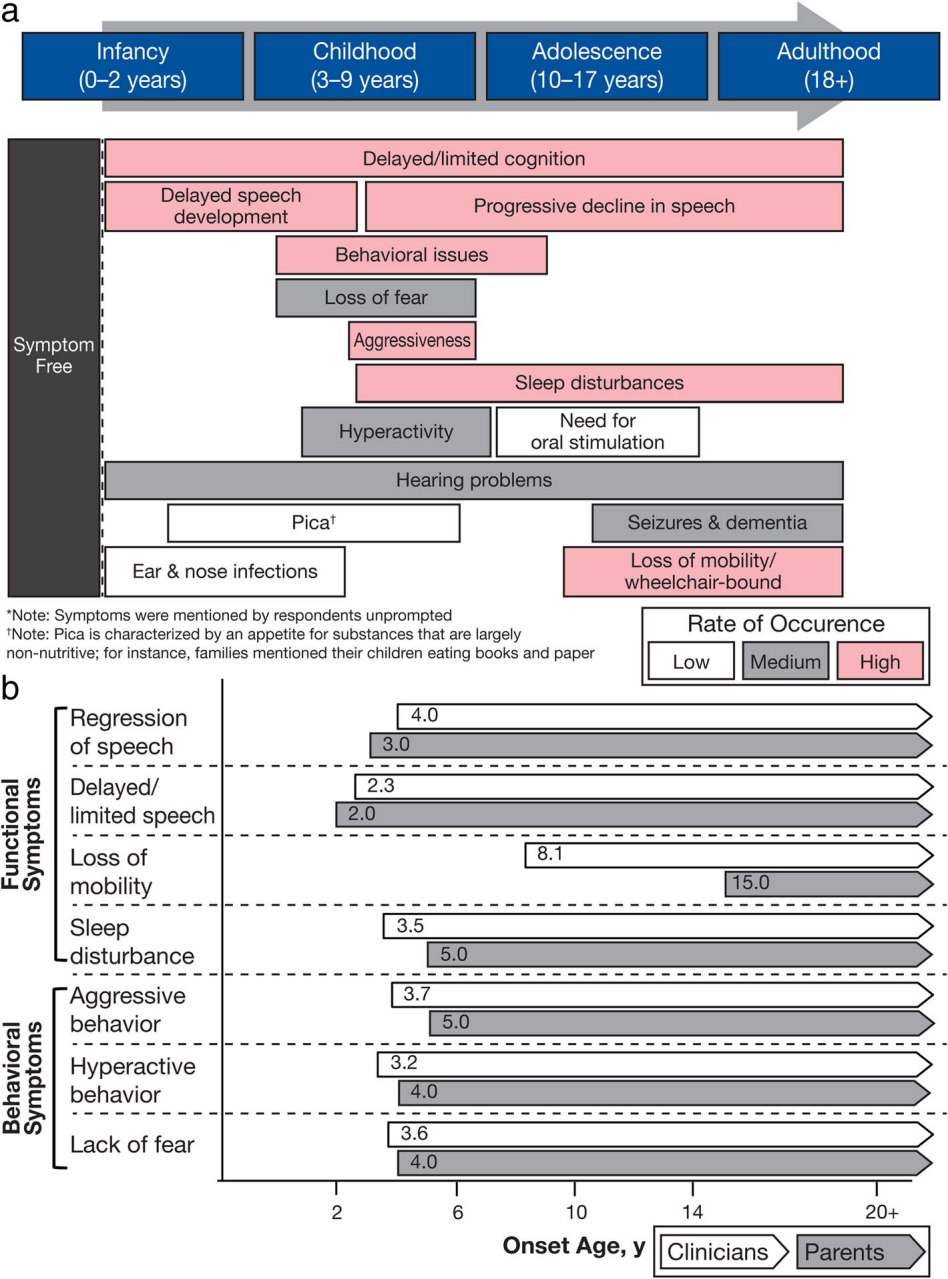
**a** Timing of Symptoms* and **b** Recognition of Physical and Behavioral Symptoms by Parents and Clinicians

Caregiver burden remains high throughout the life of the affected child but changes as symptoms progress (Fig. 1a). The average time from the appearance of initial symptoms to a confirmed diagnosis was 3 years (range, 1–9 years), with an average age at diagnosis of 4.8 years (range, 1.5–12 years). The major sources of impact on caregiver burden were the emotional impact of coping with the disease on a daily basis, associated behavioral factors (especially ‘aggressiveness’, hyperactivity, impulsiveness, and sleep disturbances), and communication difficulties with the child. Clinicians recognized physical symptoms such as loss of mobility and sleep disturbances in patients about 7 and 2 years earlier, respectively, than parents. Behavioral symptoms such as ‘aggressiveness’, hyperactivity, and lack of fear were recognized by clinicians about 1 to 2 years earlier than parents. In contrast, parents recognized the onset of speech deficits in their child before clinicians (Fig. 1b). Other important sources of caregiver burden were impact on the ability to work and financial stability, physical demands of providing care, QoL of caregivers, and impact on the family.

The psychological stress and physical exhaustion experienced by parents due to the continual caregiver burden leads to feelings of isolation and depression that are reinforced by the child’s progressive loss of acquired skills as the disease progresses.

#### Panel findings

The disease progression and burden timelines identified during the secondary research were more protracted compared with the panel’s experience. In the panel’s experience, diagnosis of Sanfilippo B is rarely made at infancy rather characteristic clinical features are generally recognized between age 1 and 8 years, hyperactivity peaks at 3.5 to 4 years, and key skills tend to stop developing at an earlier age (ie, from 3 years onward).

An early diagnosis can help reduce the initial caregiver burden; however, the rarity of the disease and associated subtle phenotypes mean that patients often encounter multiple physicians before being diagnosed. Overlooked screening triggers identified by the panel that should be recognized in correctly diagnosing Sanfilippo B include a slowing in and halting of development; the loss of acquired skills; and physical symptoms including subtle features of dysmorphia (including macrocephaly), joint stiffness, and hirsutism (especially in eyebrows). In addition, tests such as enzymatic testing, urine tests for glycosaminoglycans, and genetic testing are also important in enabling a correct diagnosis.

A broader awareness of Sanfilippo B within the global medical community is needed to lessen the major sources of caregiver burden, particularly with regard to the behavioral and functional symptoms of Sanfilippo B. Age and disease stage of the patient, in particular, determine the severity and impact on QoL of caregivers and the family. The panel agreed that the perceived aggressive behavior of the child may be better described as “physical impulsiveness” and is often misunderstood by the general public. Importantly, the lack of intentionality of the child’s behavior is recognized and shared by parents and panel members. Parents may seek to protect their child from public scrutiny and avoid situations that many engender criticism of their parenting skills.

Overall, the panel agreed that the following are important in lessening the burden of behavioral and functional symptoms on caregivers: ability of the child to communicate broadly and functionally with parents, clearly defined criteria for measuring treatment effects, and availability of treatment.

### The impact of caregiver burden on the QoL of Sanfilippo B families

#### Background research findings

**Fig. 2:**
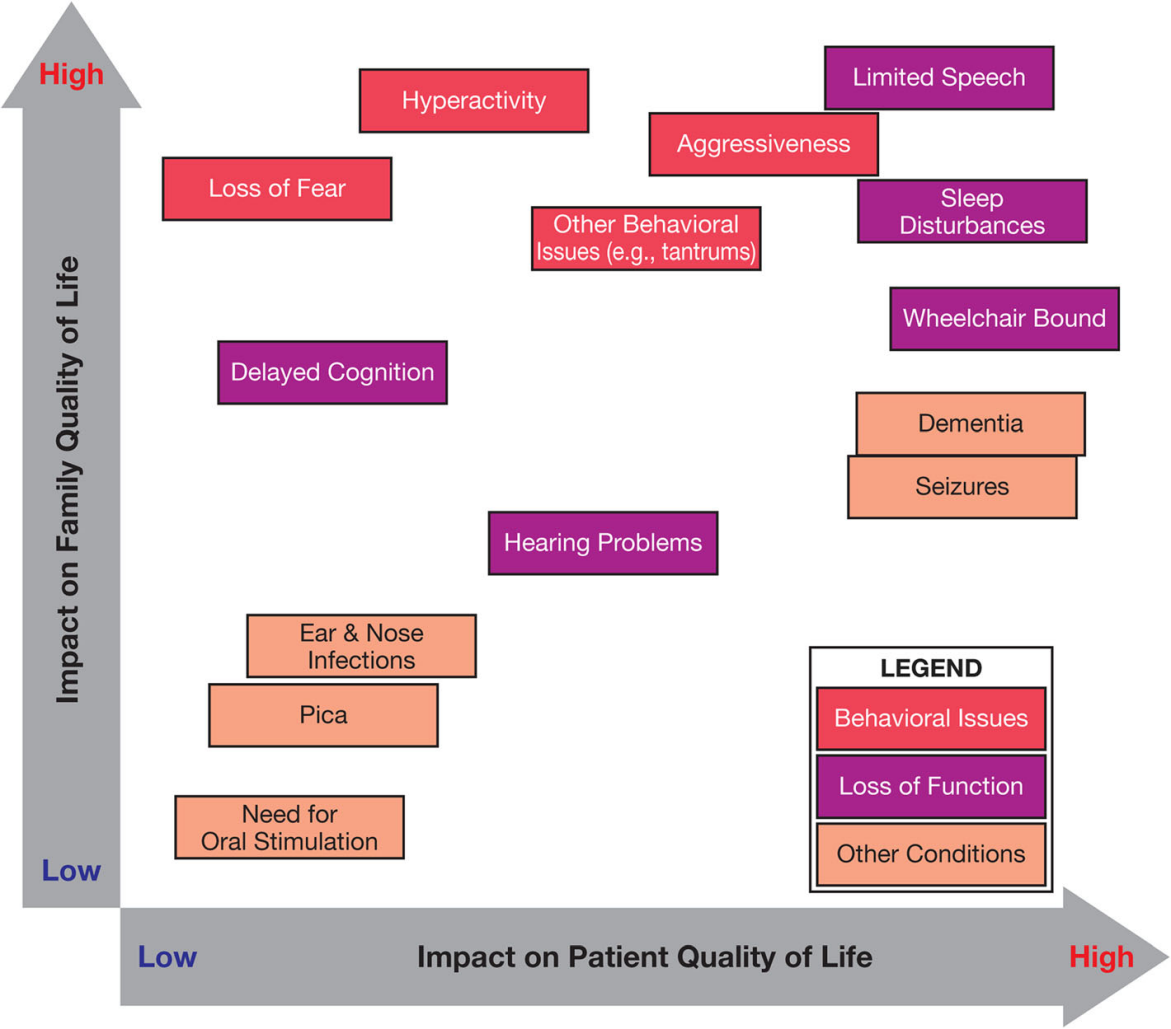
Impact of Symptoms and Behaviors on Quality of Life of Child and Family

The daily responsibilities of the caregiver leave little time to focus on anything else (Table 2). The impact of Sanfilippo B on the overall QoL of caregivers is influenced by emotional, social, and professional elements. The emotional impact on caregivers is driven by psychological stressors (eg, anxiety, depression) and cumulative physical exhaustion associated with caring for the patient. Families are often restricted in doing normal activities, which leads to feelings of social isolation. Career development and professional aspirations are often halted or limited, and financial concerns may become an issue. The level of impact on the QoL of the child and family will differ depending on the age, symptoms, and behavior of the patient (Fig. 2).

**Table 2:** Responsibilities of the Caregiver

Dealing With Behavioral Issues	
Aggressiveness^a^	Parents need to be on constant watch for potential people (eg, other children or elderly) that their child may harm
Loss of fear	Because children with Sanfilippo B are delayed cognitively, they do not recognize the inherent dangers of • Crossing the street without an adult • Walking into the deep end of a pool (and not being able to swim) • Putting their hands into very hot water • Eating anything that they can reach
Hyperactivity	Hyperactive children physically exhaust parents, who may need to chase after them going from room to room or keep them out of harm’s way
Day-to-Day Functioning	
Sleep issues	Waking up in the middle of the night several times to check on the child and/or having the affected child wake up the parents due to child’s sleep disturbance can have a cumulative detrimental impact on the parents’ ability to function
Transportation	Getting children to and from doctor’s appointments and into cars (staying still)
Medication administration	Ensuring that children receive medications when needed can be particularly challenging when their children do not follow directions
Bathing/grooming/toilet/dressing	General grooming/hygiene and toilet duties like bathing, brushing teeth, and brushing/combing hair
Food preparation	Preparing the majority of meals at home because going out is generally considered troublesome
Mobility assistance (wheelchair)	Helping the child get around; climbing staircases can be particularly tiring, especially if wheelchair transportation is involved

^a^The term aggressiveness refers to perceived disruptive behavior

#### Panel findings

**Fig. 3:**
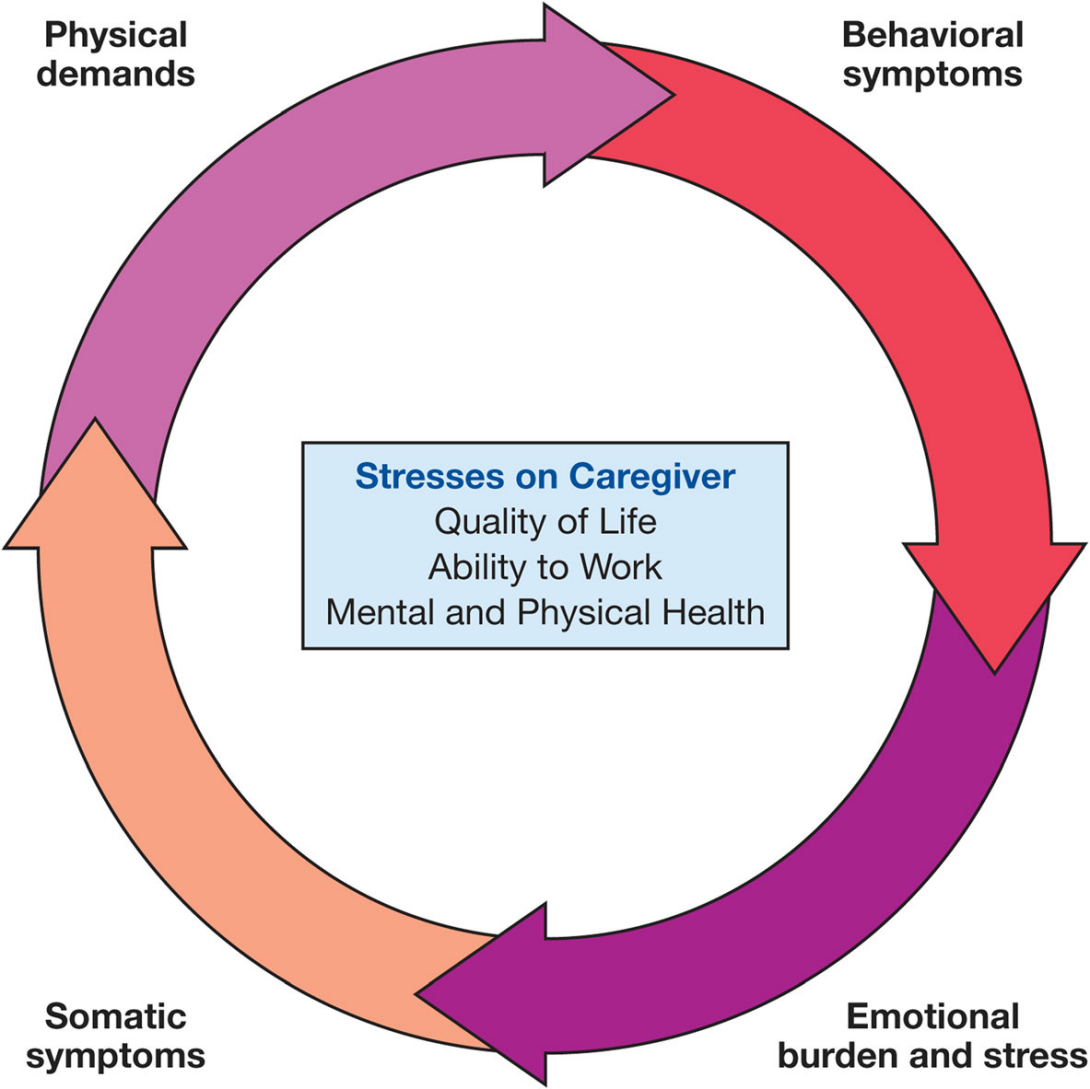
Caregiver Stress Cycle. As the patient ages, the level of burden remains high, but requirements of care change as the disease progresses. Parents and caregivers experience new stresses as they encounter different symptoms and behaviors. The stress cycle of caregiver burden repeats as caregivers readjust to the changing needs of the patient, which has a detrimental effect on the quality of life of the caregiver

There is a continuum of symptoms and behaviors that have a cumulative impact on caregiver burden and the QoL of families. The changing care needs of the patient lead to a “stress cycle” associated with the emergence and evolution of symptoms and associated needs of the patient as the disease progresses (Fig. 3).

Caregiver burden can fluctuate depending on day-to-day circumstances, such as poor sleeping patterns or stressful environments that exacerbate behaviors. For example, recognition of sleep disturbances may be due to differences in definition and may be noted by parents before clinicians. Because the child’s sleeping pattern cannot be broken, parents may normalize this behavior and often sleep with their child, as this may lead to decreased stress.

Neurologic abnormalities present additional challenges such as mood problems (eg, episodes of distress triggered by specific incidents), but these are often time-limited, differ by the phase of the disease, and may be viewed as more intense at onset by siblings and other relatives. Communication between the parent and child can also be challenging. Although other forms of communication beyond speech are available, parents may not be aware of these services or be able to justify the child’s need for such interventions to therapists or insurance providers.

Disease progression can be rapid, and parent expectations can change; thus, physicians must provide realistic expectations regarding the impact on the patient and family. In general, caregiver burden shifts over time from managing behavioral issues to the provision of physical and medical care as the skills acquired by the child are lost. The impact this has on families is often overlooked and may be displayed via compensatory coping mechanisms (eg, carrying the child or limiting social engagements). Siblings may receive less attention from their parents, resulting in less communication with parents and a feeling of social isolation. The long-term effects on siblings is unclear. Overall, the panel agreed that given the burden placed on caregivers and families, early counseling, mental health screening of parents and family members, and a support network are vital.

### Mitigating disease burden

#### Panel findings

A number of supportive measures could be put in place to mitigate caregiver burden (Table 3). The panel agreed that many of these supportive measures should be introduced early to set realistic expectations and minimize the emotional and physical burden associated with the disorder.

**Table 3:** Supportive Measures That Can Mitigate Caregiver Burden

Support Measure	Benefit for Patient and Caregiver	Outcome Measure
Counseling services	Better understand disease course Manage expectations Early introduction to quality palliative care	Defined acceptable outcome for the patient and their families
Adjunctive treatment	Alleviate somatic symptoms, including sleep disturbances and gastrointestinal symptoms	Reduced physical burden Reduced adverse events with predictable management Reduced nonattendance at school and work Cost savings
Psychiatric support	Manage/support emotional stresses, anxiety, and depression	Less impact on social services Allow caregivers to effectively perform duties
Interdisciplinary clinical network	Promotes integration and coordination of care Streamlines hospital visits and investigations Promotes early engagement with support organizations Actively monitors QoL of patients and caregivers	Reduced caregiver stress Improved productivity/time management
Support network/parent groups and patient associations	Relief from physical tasks Sharing of experiences Exchange of information on support services	Reduced emotional and physical burden
Financial assistance	Supports any potential loss of income Facilitates patient travel and home modifications	Reduced emotional, social, and professional burden
Provision of respite and palliative care	Allows caregivers time to themselves Allows time for planning and assessment of the value of continual and quality care	Reduced emotional and physical burden

*QoL* quality of life

### Panel recommendations

#### Development of a Sanfilippo-specific QoL questionnaire

Although generic caregiver and patient questionnaires have been used to assess various forms of mucopolysaccharidosis [9, 19–21], a Sanfilippo-specific QoL questionnaire is needed to help better align caregiver and clinician assessments of caregiver burden. This could be a generic Sanfilippo-specific QoL questionnaire as Sanfilippo B differs little from type A. Specific QoL questionnaires have been developed for other degenerative neurologic disorders in adults such as Alzheimer disease [22, 23] and Huntington disease [24, 25]. Additionally, the direct and indirect economic impact of conditions such as Dravet syndrome [26] or tuberous sclerosis [27] may be useful in guiding the development of a Sanfilippo-specific QoL question, given that these conditions are associated with similar issues (eg, multiple comorbidities, reduced QoL for patient and caregiver, reduced work productivity for caregiver). The questionnaire would allow clinicians to better understand the effects on caregivers in terms of their emotional, physical, mental, and social well-being and the impact that care has on the financial status of the caregiver. Development of a questionnaire presents an opportunity to properly define the aspects of the disease that most affect QoL. A phase-dependent series of questions broken down by age and phenotype is desirable. Furthermore, qualitative and quantitative measurements could be ascribed to particular symptoms or behaviors, such that patient symptoms and response to treatment can be assessed. Although capturing normative data and validation would be challenging, information obtained from such a questionnaire would help guide treatment decisions as the disease progresses.

#### Clinical best practice

Clinicians should develop good relationships with the families of Sanfilippo B patients through direct, regular personal contact. Such interaction should include counseling and a full explanation of the patient’s journey at initial diagnosis; management of caregiver expectations regarding diagnosis, symptoms, caregiver burden, and disease progression; and early introduction of quality palliative and respite care. Although management of patients with Sanfilippo B varies by country, the development of an interdisciplinary clinical network that promotes integration of care should be the standard of care. The clinical network can assist in streamlining hospital visits and laboratory investigations, promoting early engagement with support organizations to assist families, and actively monitoring QoL of patients and caregivers, with the ability to initiate referrals for depression screenings where appropriate. Ultimately, treatment guidelines are needed.

#### The role of treatment in alleviating caregiver burden

Curative treatment is the preferred solution; however, this is not currently a viable option. Although there are a number of different treatments currently under development, the panel believes that treatments that slow disease progression are equally as valuable as treatments that maintain function. As with other progressive neurologic diseases for which no curative treatment is currently available, treatment goals for Sanfilippo B should be focused on the following 3 areas: (1) slowing disease progression and cognitive decline, as reversal of symptoms are not expected, (2) improving the QoL for patients and caregivers, and (3) treating physical and behavioral symptoms. Palliative support is vital, regardless of whether treatment halts disease progression.

## Discussion

This report identifies and describes the primary, and potentially modifiable, caregiver needs for patients with Sanfilippo B and illustrates the impact of these needs on the burden among caregivers. The research findings presented here and to the clinical advisors panel were based on a small sample of clinicians and caregivers and a recall bias may, in part, account for differences in experiences of clinicians versus the medical record.

Caregiver burden is dynamic and is impacted by the age and disease stage of the patient. As a result, caregiver burden remains high throughout the patient’s life, with caregivers experiencing a disease-specific and predominantly negative impact on QoL. The research findings revealed differences between the timing of symptom recognition by parents compared with clinicians. Loss of mobility was recognized much earlier by clinicians than parents, who may take pragmatic compensatory steps to cope (eg, carrying the child) with the situation; however, there is currently no consistent definition used by clinicians or caregivers as to what constitutes loss of mobility. Clinicians also recognized disturbed sleeping patterns in patients earlier than parents, who may manage this by adapting and normalizing their sleep patterns to decrease stress. As shown in Fig. 2, the impact of seizures on patient QoL was high but affected family QoL to a lesser degree. This moderate effect of seizures on family QoL was similar to that reported in studies of epilepsy and other less prevalent long-term neurologic conditions [28]. The lesser impact that seizures has on family QoL may relate to the episodic nature of these traumatic events compared with the overall impact of the physical demands and stress of coping with the daily needs of the patient.

## Conclusion

The panel findings indicate that to alleviate caregiver burden, a wide range of Sanfilippo B–specific interventions and support services should be provided that not only target the behavior and symptoms of the patient but are also sensitive to the changing needs of the caregiver’s burden as the disease progresses and provides support to the caregiver’s mental and physical health needs. The panel recommends development of a Sanfilippo-specific QoL questionnaire and specific guidance on best clinical practice for patients with Sanfilippo B, as well as recognition of the important role treatment has in alleviating caregiver burden.

## Additional file

Additional file 1: Mucopolysaccharidosis IIIB Research Discussion Guide. Complete questionnaire used for the secondary background research that the clinical advisors used to inform their discussion and recommendations. (PDF 447 kb)

## Data Availability

The dataset supporting the conclusions of this article are provided in this article and no additional data are available to post to any repository.

## Abbreviations

MPS III: Mucopolysaccharidosis type III
QoL: Quality of life
