# Supplementary material for: Analysis of the caregiver burden associated with Sanfilippo syndrome type B: panel recommendations based on qualitative and quantitative data
Source: Orphanet J Rare Dis. 2019 Jul 8;14:168. doi: 10.1186/s13023-019-1150-1 (PMC6615275; doi:10.1186/s13023-019-1150-1)
Supplement: Supplementary file 1 — Mucopolysaccharidosis IIIB Research Discussion Guide. Complete questionnaire used for the secondary background research that the clinical advisors used to inform their discussion and recommendations. (PDF 447 kb) [file 13023_2019_1150_MOESM1_ESM.pdf]

MPS IIIB Family Research Discussion Guide

Audience: MPS IIIB Family Members
Interview Length: 60 minutes

**Discussion Guide and Objectives (for Moderators Only)**
**Objectives:** Interviewer plans to conduct ~20-30 interviews with family members who are currently caring for patients with MPS IIIB (Sanfilippo Syndrome Type B). The focus of this research will be to identify the patient journey for MPS IIIB patients and the issues their families face.<br><br>**Interview Flow:**<br>1. **Introduction and Background** (~3-5 minutes)<br>2. **Patient Journey** (~28-30 minutes)<br>3. **Clinical Program Considerations** (~15-17 minutes)<br>4. **Unmet Needs & Product Expectations** (~5-6 minutes)<br>5. **Wrap-up** (~1-2 minutes)<br>*** Priority sections / questions to be addressed in interviews. Other secondary questions and learnings are noted as "time permitting."**<br><br>Please note that the discussion guide is meant to serve as a starting point for discussion, not a script. The moderator will use each individual respondent's experiences and opinions to tailor the interview.

Section 1: Introduction and Background (~3-5 minutes)

Purpose: to build rapport, ease respondents into the interview, and learn about his/her background, demographics, current family relationships and support / resources

Thank you for taking the time to speak with me today. We are scheduled for a 60-minute discussion. Is this still a good time for you?

We are currently working to better understand what is like to take care of a family member with MPS IIIB. We appreciate your time and sharing your story with us.

Our discussion should take about 60-minutes and I want to reiterate that there are no "right" or "wrong" answers; any insight you are willing and comfortable to provide will be extremely helpful for our understanding. Please note, that this research is being sponsored by a healthcare company, however, everything we talk about today will be anonymized and confidential.

I was hoping to tape our conversation in order to facilitate note taking, is this ok with you?
I also want to assure you that your identity is being protected. Is that okay?

The purpose for this interview is to learn your experience taking care of your loved one with MPS IIIB. We want to understand the support and resources you have, and any challenges and issues you face while taking care of your child over time. Once we get through some background and diagnosis info, we will go right into learning about your experiences taking care of your child.

Do you have any questions before we begin?

I would like to start by learning about you…

1. Could you please start by telling me a little bit about yourself?
   Listen for and probe on the following:
   a. Geographic region (urban, suburban, rural)
   b. Ethnicity
   c. Family life (e.g., marital status)
   d. Total number of children
   e. Profession (if working)

2. Now, I'd like to know a little about the patient with MPS IIIB.
   a. What is your relationship with the patient?
   b. How old is your **[SON, DAUGHTER, ETC.]**?
   c. How old was your **[SON, DAUGHTER, ETC.]** when he/she was diagnosed with MPS IIIB?
   d. Does your **[SON, DAUGHTER, ETC.]** have any other conditions besides MPS IIIB? Which ones? **[*Moderator note: briefly go over this to assess overall health / comorbidities*]**
   e. Who else is a part of your family?
   f. Who are you and your **[SON, DAUGHTER, ETC.]** currently living with?

Section 2: Patient Journey (~28-30 minutes)

Purpose: Explore how MPS IIIB has affected the lives of the child and *the family, characterize their journey from presentation to potential treatment considerations, identify the general timeline for each key phase in the journey, and identify challenges facing them / how they navigate these challenges*

Patient Journey: Baseline Developmental Milestones

3. I would like to understand your **[SON, DAUGHTER, ETC.]** a little bit better, starting before their diagnosis of MPS IIIB.

   When did your **[SON, DAUGHTER, ETC.]** achieve the following skills? ***[Moderator note: Probe accordingly based on age]:***
   a. Develop speech
      i. Start speaking first word
      ii. Start speaking full sentence

b. Start walking
c. Start school (if appropriate)
d. Other milestones achieved during your **[SON, DAUGHTER, ETC.]'s** growth **[Unaided]**

Patient Journey: Diagnosis

4. When did you first become concerned about your **[SON, DAUGHTER, ETC.]'s** development? What were those issues?
   [Moderator note: Moderator allow open-ended response first and probe on the following if not mentioned. Key symptoms will be discussed in detail later on after an understanding of the journey to a confirmed MPS IIIB diagnosis (if applicable)]:
   a. Cognitive (cognitive decline, progressive loss of skills, seizures, etc.)
   b. Behavioral (aggressive behavior, hyperactivity, loss of fear, etc.)
   c. Functional (loss of: speech, hearing, vision, mobility, inability to sleep for prolonged period of time, etc.)
   d. Other issues (unaided)

5. What was / were the trigger(s) that led to the first doctor visit?

6. Now, let's focus on your experiences with doctors starting with the first doctor seen.

   Initial Doctor Visit
   a. How old was your **[SON, DAUGHTER, ETC.]** when you first took him/her to see a doctor?
   b. Which doctors were first seen / consulted?
   c. What were the results from these visits?
   d. What was / were the original suspected disease(s) (if any / aware)?
   e. Do you recall any diagnosis test(s) that were performed?
      i. If yes, what was / were the diagnosis test(s)?
      ii. If yes, what were the diagnosis results?
         1. Did these results confirm MPS IIIB?
         2. If yes, how long did you have to wait until get the diagnosis result(s)?
   f. What plan / approach was recommended by the doctor, if any?
   g. Did you get referred to any other specialist(s)?
      i. If yes, which specialist(s) you were referred to?
   h. Was / were there any follow-up doctor visit(s) after this visit?

7. Now let's focus on your **[SON, DAUGHTER, ETC.]'s** experiences with MPS IIIB diagnosis **[If initial doctor mentioned in Q.6 did not provide MPS IIIB diagnosis]**:

   Doctor who confirmed MPS IIIB Diagnosis
   a. What type of doctor provided the MPS IIIB diagnosis?
   b. When / at what age was the MPS IIIB diagnosis confirmed?

c. How many doctors were consulted before MPS IIIB was confirmed? What type of doctors were consulted before? What were the conclusions coming out of these doctors?
d. Do you recall any diagnosis test(s) that were performed by this doctor who confirmed MPS IIIB diagnosis?
   i. If yes, what was / were the diagnosis test(s)?
   ii. If yes, what were the diagnosis results? Did these results confirm MPS IIIB?
   iii. If yes, how long did you have to wait until get the diagnosis result(s)?
e. How did the doctor confirm MPS IIIB diagnosis? ***[Moderator Note: Moderate to listen for and probe on if there were other factors that helped with the diagnosis other than diagnostics / tests]***
f. How long did it take the doctor to confirm MPS IIIB? Was MPS IIIB suspected for a period of time before finally confirmed?
g. About how long did it take from the emergence of the first symptom / concern to a confirmed diagnosis of MPS IIIB? About how long did it take from the first doctor visit to a confirmed diagnosis of MPS IIIB?
h. Was / were there any second opinion?
i. What plan / approach was recommended by the doctor, if any?
j. What kind of treatment / medication was recommended by the doctor, if any?
k. After confirming MPS IIIB, were there any follow-up doctor visits?

8. Now let's focus on your **[SON, DAUGHTER, ETC.]'s** experiences with his / her **current doctor**:

   Current Doctor Visits after MPS IIIB Diagnosis

   a. After the MPS IIIB diagnosis, which doctor did you and your **[SON, DAUGHTER, ETC.]** see?
      i. **[Only ask If different from doctor who provided the diagnosis]**:
         i. What kind of doctor(s) did you see after MPS IIIB diagnosis?
         ii. Why did you switch to this doctor?
   b. How often do you see this doctor?
   c. Did the plan / approach / treatment change with this doctor?
      i. If yes, why and how did the doctor adjust the plan / approach / treatment?

9. How did the diagnosis of MPS IIIB make you and your family feel?
   a. Now I want you to do a small exercise with me. I will read a sentence and please fill in the blank:
      i. "The diagnosis of MPS IIIB means _______ for my **[SON, DAUGHTER, ETC.]**."
      ii. "The diagnosis of MPS IIIB means _______ for me and my family."

10. Was there anyone you turned to for emotional, caretaking, and/or financial support after learning of the diagnosis?
    [Moderator note: Moderator allow open-ended response first and probe as necessary]:

   a. Family members
   b. Friends
   c. Patient support groups
   d. Patient community (online, local, etc.)
   e. State / Government funding
   f. Healthcare organizations

11. Which **sources of information** do you most frequently use to learn more about MPS IIIB and how do you use them? ***[Moderator note: Moderator allow open-ended response first and probe on the following if not mentioned; moderator to capture all sources and how do respondent use each resource]:***
    a. Patient support groups, (specify)_______
    b. Patient communities (online, local, etc.), (specify)_______
    c. Doctors / specialists, (specify)_______
    d. Healthcare organizations, (specify)_______
    e. Online resources / websites, (specify)_______

Patient Journey: Symptom Progression and Impact

12. What developmental challenges (e.g., delays in and/or loss of mental development, motor skills, etc.) does your **[SON, DAUGHTER, ETC.]'s** currently experience? ***[Moderator note: Moderator allow open-ended response first and probe on the following if not mentioned]****:*
    a. Cognitive (cognitive decline, progressive loss of skills, seizures, etc.)
    b. Behavioral (aggressive behavior, hyperactivity, loss of fear, etc.)
    c. Functional (loss of: speech, hearing, vision, mobility, inability to sleep for prolonged period of time, etc.)
    d. Social issues (e.g., perceived social stigma, etc.)
    e. Other key developmental challenges (unaided)

13. When did each of these developmental challenges first occur? How have these developmental challenges progressed / gotten worse over time?
    a. *How often do these developmental challenges get worse? Does this vary based on age / disease progression?*
    b. *Listen for and probe on the frequency in which developmental challenges occur or get worse and whether that frequency is consistent over the course of disease progression.*

14. How have these developmental challenges impacted your **[SON'S, DAUGHTER'S, ETC.]** growth / skills development?

15. How have these developmental challenges impacted you and your family? ***[Moderator note: Moderator allow open-ended response first and probe as necessary]:***

a. Impact on family structure
b. Resultant changes in your and your family members' lives (career, family life, etc.)
c. Resultant changes in your lifestyle and that of your family members'

Key Developmental Challenges	Timing / Age of Developmental Challenges Emergence	Impact on Child	Impact on Family members
**Cognitive** (cognitive decline, progressive loss of skills, seizures, etc.)			
**Behavioral** (aggressive behavior, hyperactivity, loss of fear, etc.)			
**Functional** (loss of: speech, hearing, vision, mobility, inability to sleep for prolonged period of time, etc.)			
**Other** key developmental challenges (unaided)			

16. **[IF CHILD HAS A SIBLING]** How would you characterize the development of your **[SON, DAUGHTER, ETC.]** with MPS IIIB compared to your other child without MPS IIIB?

Patient Journey: Symptom Management

17. Now we want to talk about your current methods used to manage your **[SON, DAUGHTER, ETC.]'s** condition. ***[Moderator note: Listen for and probe on more/fewer medications, more/less frequent visits to doctors, which management seems to work/not work]:***
    a. What type of treatment / approaches you use?
    b. What and how many medication(s) is your **[SON, DAUGHTER, ETC.]** currently taking?
    c. What alternative approaches (e.g., yoga, meditation, or Eastern medicine) have you tried?
    d. How frequently do you take your **[SON, DAUGHTER, ETC.]** to go see a doctor?
    e. Which type(s) of doctor(s) / specialist(s) do you typically go to, if different from who you see today?
    f. How has each of the following changed over time (if at all)?
        i. Treatment / approaches
        ii. Frequency of doctor visit
        iii. Type of doctor consulted

18. What are the treatment options (if any) considered? Which ones have you tried? How do you feel about them?
    Listen for and probe on:
    a. Bone marrow replacement
    b. Stem cell transplantation
    c. Enrollment in clinical trials / investigational drugs
    d. Any other (unaided)

19. Which doctors recommended these treatment options (if any)?

*Current Life: Major Issues and Day-to-Day Challenges**

20. Now, I want to more broadly understand what impact the developmental challenges may have on your child.

    What are the major issues your **[SON, DAUGHTER, ETC.]** currently face as a result of the earlier mentioned developmental challenges? ***[Moderator note: Moderator allow open-ended response first and probe as necessary]:***
    a. Social issues / challenges (e.g., feelings of isolation, difficulty making friends, etc.)
    b. Academic issues / challenges (e.g., whether child can attend a school, cannot keep up with school work, learning disabilities, etc.)
    c. Psychological issues / challenges (e.g., aggressiveness, depression, etc.)
    d. Emotional issues / challenges (e.g., mood swings, temper tantrums, etc.)

21. How do these issues impact your **[SON, DAUGHTER, ETC.],** you and your family? *Listen for and probe on whether child can attend school.*

22. How have these issues and challenges changed over time, if at all?

23. Now let's focus on challenges for **you and your family**. What would you say are the major issues or challenges you and your family currently face due to taking care of your **[SON, DAUGHTER, ETC.]**? ***[Moderator note: Moderator allow open-ended response first and probe as necessary]:***
    a. Social issues / challenges for you and your family
    b. Career challenges for you and your family member(s)
        i. Whether you or your family member(s) had to give up work to provide care
    c. Financial challenges for you and your family:
        i. Whether additional financial aid from government is provided
    d. Psychological issues / challenges for you and your family member(s)
    e. Emotional issues / challenges for you and your family member(s)
    f. Family relationship issues / challenges:
        i. **[IF RESPONDENT IS MARRIED]** What impact – if any – does taking care of someone with MPS IIIB have on your relations with your spouse?

24. How do these issues and challenges impact your **[SON, DAUGHTER, ETC.],** you and your family?

25. How have these issues and challenges changed over time, if at all?

26. How do these challenges impact your **[SON, DAUGHTER, ETC.]**, you, **[HIS/HER]** siblings (if any), and other family members?

    a. Do your children play together or do they need to be separated? How does that make you feel?

27. How have these challenges and impact evolved over time, if at all?

28. Thinking of these issues and challenges, which of these do you consider to be a **major turning point** for you and your family? Why? *Listen for and probe on key trigger points of change for family.*

29. Thinking about everything we just discussed, what are the **top 3 pain points, frustrations, challenges, or issues**? What impact do these have on you and your child's quality of life?

30. What kind of support, help and resources you are currently utilizing to take care of your **[SON, DAUGHTER, ETC.]** (e.g., nursing support, home care support, etc.)?

31. Now I want you to do a small exercise with me. I will read a sentence and please fill in the blank:
    a. "Living with MPS IIIB means _______ for my **[SON, DAUGHTER, ETC.]**"
    b. "Taking care of someone with MPS IIIB means _______"

Section 3*: Clinical Program Considerations (~12-17 minutes)

Purpose: Explore treatment considerations in detail and leverage respondent's prior experiences to help identify tactics to best have **[SON, DAUGHTER, ETC.]** *sit through therapy involving needles*

32. How difficult is it to bring your **[SON, DAUGHTER, ETC.]** to a doctor's office? What is required to bring **[HIM/HER]** to a doctor's office?
33. About how long can your **[SON, DAUGHTER, ETC.]** sit still?
34. What method(s) and technique(s) have you used that work best to keep your **[SON, DAUGHTER, ETC.]** to sit still? *Listen for and probe on what happens during dentist visits.*
    a. **[ONLY ASK IF son/daughter is > 6 years old]** Thinking back when your **[SON, DAUGHTER, ETC.]** was 6 years old or younger, what method(s) and technique(s) did you use that work best to keep he/she to sit still back then?
35. Did your **[SON, DAUGHTER, ETC.]** have any past experience when **[HE/SHE]** had to deal with medical procedures requiring a needle (e.g., getting blood drawn)?
    What was the procedure like and what did you do to help make the process smooth?

36. Now I will describe a hypothetical scenario to you, please note that this is not specifically about your **[SON, DAUGHTER, ETC.]**:
"A child with MPS IIIB needs to go to a doctor's office and sit still while having a therapy delivered through a needle and tube." Please think about this hypothetical scenario and let us know:
    a. Would this hypothetical scenario be a barrier to getting this treatment for your **[SON, DAUGHTER, ETC.]**?
    b. **[ONLY ASK IF son/daughter is > 6 years old]** Thinking back when your **[SON, DAUGHTER, ETC.]** was 6 years old or younger, would this hypothetical scenario be a barrier to getting this treatment for your **[SON, DAUGHTER, ETC.]**? Why / why not?
    c. What is the realistic length of time your **[SON, DAUGHTER, ETC.]** can sit still under that hypothetical scenario (with or without distraction)?
    d. Is it feasible for your **[SON, DAUGHTER, ETC.]** to walk around while still having treatment administered?
    e. What are some potential issues with having treatments involving needles? *Listen for and probe for pulling at needle, keeping needle in place, etc.*
    f. What is the most manageable number of trips per week to doctor's office for you and your **[SON, DAUGHTER, ETC.]**? What about per month?

Section 4: Unmet Needs and Product Expectations (~5-6 minutes)

Purpose: Explore unmet needs for disease/symptom management and expectations for potential new therapies from the respondent's perspective

37. On a scale of 1-7 where 1 is "not at all interested" and 7 is "extremely interested", how interested would you be in new MPS IIIB therapies?
    a. How has your interest level change over time (if at all) since the initial diagnosis?

38. If there were a potential new therapy, what would be the **top three developmental challenges** (e.g., cognitive decline, hyperactivity, loss of speech and mobility, etc.) **or disease symptoms** you'd like to see improvement in? Why?
    a. Which one is the most important one? Why?
    b. Does that change over time with the severity of the disease? If so, how? *Listen for and probe on whether the top development challenge / troublesome symptom changes with disease progression.*
    c. Assuming a potential therapy can improve on this developmental challenge, but the therapy has some side effects associated with it. What kind of side effects / risks are you willing to accept for this benefit? What do you consider to be a "deal breaker"?

39. Using a potential new therapy, which **top three day-to-day challenges** (e.g., hard to take the child to doctor, sleep disturbance, social stress, etc.) would you like to see improvement in? Why?
    a. Which one is the most important? Why?

40. What is the most important progression stage you would like to delay? Why?

41. Now I will read a sentence and please fill in the blank:
    "The ideal treatment for MPS IIIB would mean ___________".

42. What are the top three benefits of a new therapy that would have the greatest impact on the quality of life for your **[SON, DAUGHTER, ETC.]**, you and the family?
    a. Of the three benefits, which is the top benefit that would have the greatest impact on the quality of life for your **[SON, DAUGHTER, ETC.]**, you, your family, and siblings (if applicable)?

Section 5: Wrap-up (~1-2 minutes)

43. Do you have any final comments or thoughts that we didn't touch on?

THANK AND CLOSE

Thank you for your time and insights. The experiences you shared with us is very useful and will help the research efforts in developing potential MPS IIIB therapy. The firm that arranged this call with you will be in touch regarding processing your honorarium.
